# Assessment of climate impact on vegetation dynamics over East Africa from 1982 to 2015

**DOI:** 10.1038/s41598-019-53150-0

**Published:** 2019-11-14

**Authors:** Wilson Kalisa, Tertsea Igbawua, Malak Henchiri, Shahzad Ali, Sha Zhang, Yun Bai, Jiahua Zhang

**Affiliations:** 1 Remote Sensing and Climate Change, School of Computer Science and Technology, Qingdao University, Qingdao, 266071 China; 2 Key Laboratory of Digital Earth Science, Institute of Remote Sensing and Digital Earth, Chinese Academy of Sciences, Beijing, 100094 China

**Keywords:** Climate-change impacts, Climate-change ecology

## Abstract

Located across the equator, the East Africa region is among regions of Africa which have previously known the severe vegetation degradation. Some known reasons are associated with the climate change events and unprofessional agricultural practices. For this purpose, the Advanced Very High Resolution Radiometer (AVHRR) version 3 NDVI (NDVI3g) and Climate Research Unit (CRU) datasets for precipitation and temperature were used to assess the impact of climate factors on vegetation dynamics over East Africa from 1982 to 2015. Pearson correlation of NDVI and climate factors were also explored to investigate the short (October - December) rainy seasons. The phenological metrics of the region was also extracted to understand the seasonal cycle of vegetation. The results show that a positive linear trend of 14.50 × 10^−4^ for mean annual NDVI before 1998, where as a negative linear trend of −9.64 × 10^−4^ was found after 1998. The Break Point (BP) was obtained in 1998, which suggests to nonlinear responses of NDVI to climate and non-climate drivers. ENSO-vegetation in El-nino years showed a weak teleconnection between ENSO and vegetation growth changes of croplands. Also, the analyzed correlations on NDVI data resulted to the higher correlation between NDVI and precipitation than that with temperature. The Hurst exponent result showed that about, 18.63% pixels exhibited a behavior, typical of random walk (H = 0.5) suggesting that NDVI growth changes may eventually persist, overturn or fluctuate randomly in the future depending on the drivers. Vegetation trends with sustainable (unsustainable) trends were 36.8% (44.6%). Strikingly, about 20% of the total vegetated area showed unsustainable trend from degradation to amelioration. More so, results reveal that the vegetation of the croplands (non-croplands) over East Africa changed insignificantly by 6.9 × 10^−5^/yr (5.16 × 10^−4^/yr), suggesting that non-croplands are fast getting reduced Nonetheless, the NDVI growth responses to monthly and seasonal changes in climate were adjudged to be complex and dynamic. Seasonally, the short rainy season showed the higher variability in NDVI than the long rainy season. Also, the DJF, MAM and SON seasons are strongly driven by precipitation variation effect of ENSO versus NDVI series.

## Introduction

The distribution of terrestrial ecosystems, including composition and mechanism from regional to global scales is heavily dependent on climate^1^. Vegetation cover is a very delicate and unstable portion of the ecosystem^2^. Both the phenological metrics variations and the transformations in vegetation of different land cover types over a particular region are referred to as vegetation dynamics^3^. Vegetation is an important variable in land atmosphere interactions^4^ and vegetation changes are considered by the detection of vital phenological quantities, usually the green-up as well as the maturity or the senescence and the length of season (LOS)^5^. There exist a direct connection between vegetation’s density changes, human beings health and hydro-ecological changes. This is to mean the impact of anthropogenic activities as well as natural phenomena such as an El Niño Southern Oscillation (ENSO)^6^.

Causes for vegetation dynamics largely depend on geographical conditions, including the climate constraints together with the inhabitant’s daily activities. The East African economy highly depends on exploiting natural resources; this has direct effects on the abnormally changing climate^7,8^. These changes are shown as the diversion against the normal climatology of the region, obtained by analyzing the long-term recorded data, usually over about thirty years of time^9^. Hussein^8^ reported a prediction of warming scenarios from 0.2 °C to 0.5 °C per decade across the African continent while Hulme *et al*.^10^ suggested that under such temperature conditions, equatorial East Africa will possibly indicate about 5–20% increased precipitation in DJF season and 5–10% decreased precipitation in JJA season by 2050. In particular, over East Africa, rainfalls greatly vary during the year, with wider fluctuations occurring over the years and across the two rainy seasons, resulting in socioeconomic and hydrological impacts^11^. The dry season occurs in between the two rainy seasons from June to August^12^. The rainfall modes are majorly influenced by the Indian Ocean’s monsoonal moisture flux, regulated by the north to south movement of the Inter-tropical Convergence Zone (ITCZ) over the Equator^13,14^.

In recent years, satellite sensors have become an important tool for vegetation dynamics and trends at regional to global scales^14–19^. The normalized difference vegetation index (NDVI) derived from satellite data is an important indicator that can be used to analyze live green vegetation growth condition, and reveal response of vegetation dynamic to the climate change^20–25^. In last three decades, some works are carried out from models and statistical methods which have been used to study NDVI changes in East Africa. Nicholson *et al*.^26^ evaluated NDVI-rainfall relationships in East Africa and the Sahel, and found that the spatial patterns of annually-integrated NDVI closely reflect mean annual rainfall. Pelkey *et al*.^27^ used NDVI imagery analysis to study changes in the vegetation over Tanzania and indicated the increase in greenness during 1982 to 1994. Plisnier *et al*.^28^ used NDVI, ENSO and climate data to study tele-connections between ENSO and ecosystem and their work confirmed ENSO effects on climate and ecological fluctuation. Based on the anomaly of NDVI in East (South) Africa from 1997 to 2000, there was a reversal in NDVI linked between the precipitation traces, changes from El Nino to La Nina conditions^29^.The raising temperature and weakening precipitation, which incited drought and its consequences of the reduced vegetation in Kenya and Tanzania^30^. Recently, Landmann and Dubovyk^31^ merged the NDVI data with rainfall records from the Tropical Rainfall Measuring Mission (TRMM) for 2001–2011 over East Africa; the results showed the deteriorating yields as a consequence of both the vegetation degradation and anthropogenic alterations of the land. Detsch *et al*.^12^ applied the FAO Land-cover Classification System (LCCS) over Tanzania of East Africa, and by using MODIS NDVI data from 2001 to 2005, which resulted in the NDVI profiles of different Afri-cover classes were linked to the seasonal variations of the local vegetation, and agreed with rainfall patterns, particularly in the arid zones.

Considering the existent research situation in the region of interest, the aim of the present research work is to give a more detailed and updated analysis of the changes in vegetation in relation with climate and ENSO signals over East Africa from 1982 to 2015. Most of the analyses over the region resulted from regionally averaged and short-term recorded NDVI; previous studies that used long NDVI records ended in 2011. There is a need to understand whether the updated records in AVHRR GIMMS NDVI records indicates any different relationship between NDVI and climate; how NDVI and climate variables fluctuate from their mean position in different ENSO years too. Also, studies over the region did not pay attention to spatial persistence of vegetation trends. Trends can influence data records and appear to be persistent when there is not a real relationship between the data records. Thus, the correct scaling behavior of the vegetation data series has to be well characterized. Thus, the specific objectives of this research are to: (1) study the breaking points in NDVI from 1982 to 2015; (2) analyze the seasonal, monthly and yearly time series of NDVI indices; (3) extract phenological parameters, climatic parameters for short and long rainy seasons, NDVI-climate anomalies as well as the sea surface temperature (SST) anomalies and (4) identify the spatial persistence in trends and the sustainability of the observed trends and also specify the most vulnerable areas over East Africa using NDVI.

## Study Area

**Figure 1:**
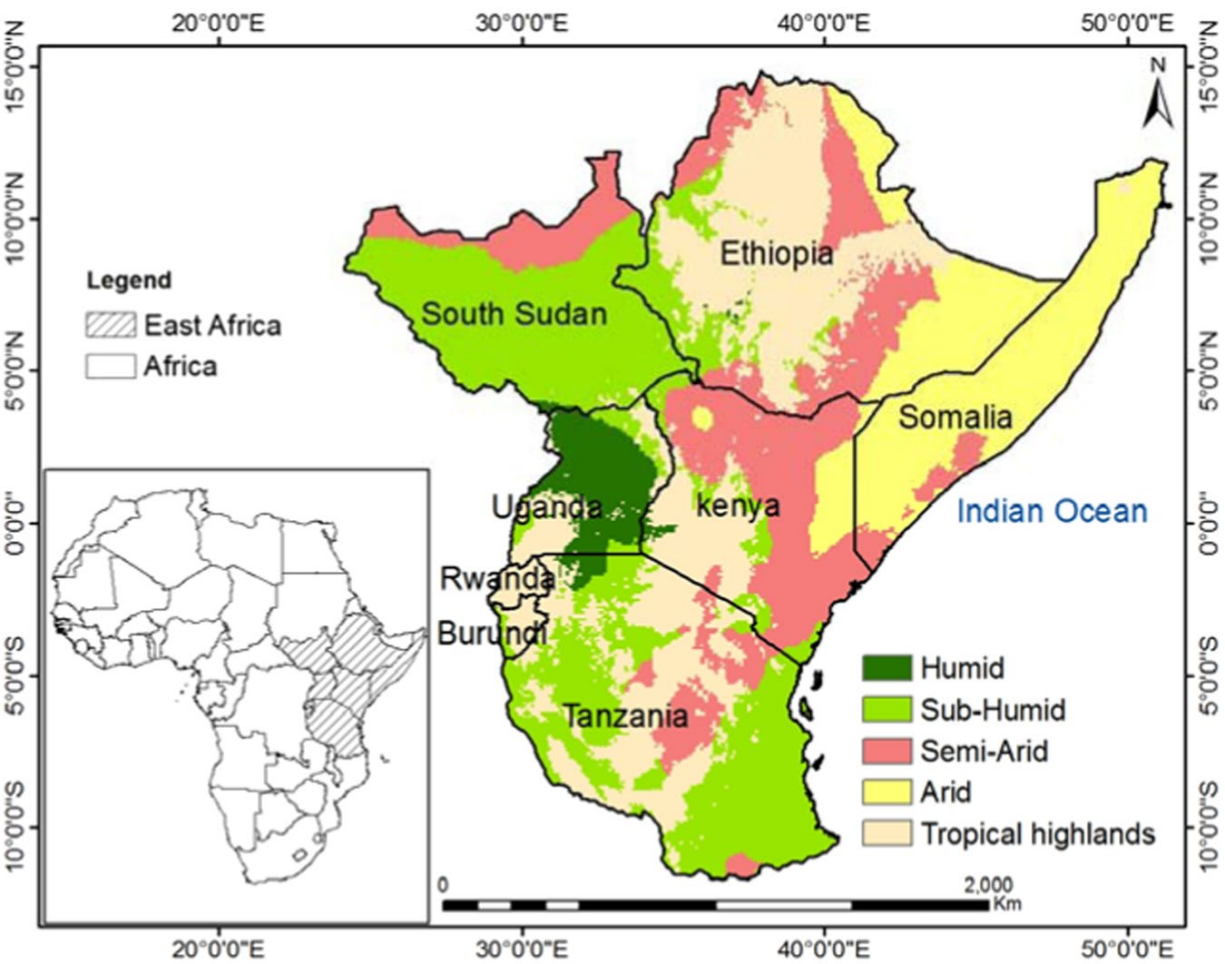
Study area (East Africa) and its location on Africa.

This study will be applied to the whole East African Community (EAC) block, comprising 8 countries namely Rwanda, Uganda, Tanzania, Burundi, Ethiopia, Kenya, Somalia and South Sudan (Fig. 1). The study area has a land mass of about 9.8 million square kilometers.

**Figure 2:**
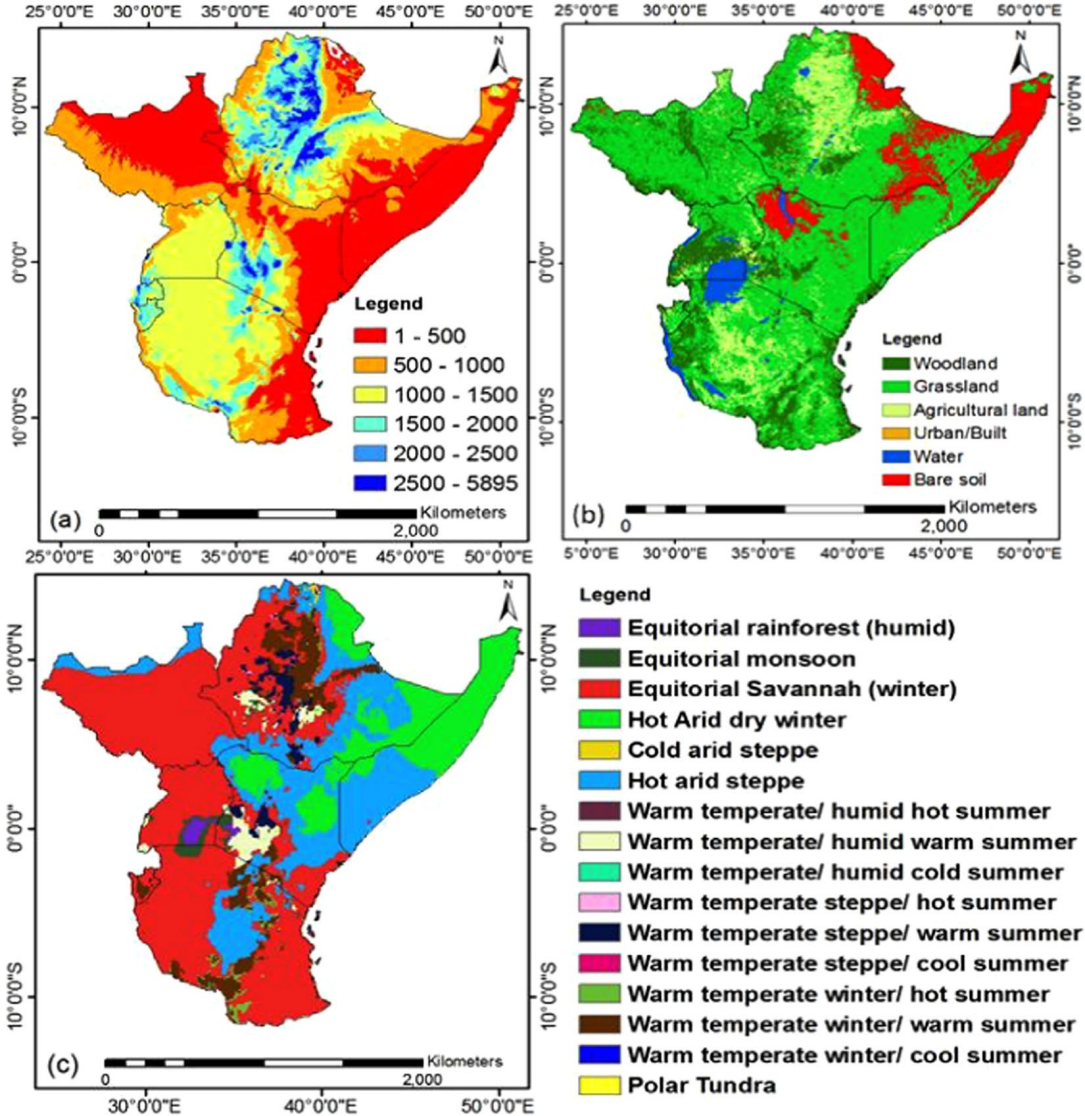
(**a**) DEM (meter) and (**b**) Land cover map and (**c**) Climate map of East Africa^37^.

A large part of East Africa experiences two distinct rainfall seasons: “long rains”, which extend during March–May (MAM), and a season with “short rains”, which lasts from October to December (OND). These seasons are linked to the movement of the Intertropical Convergence Zone (ITCZ) northward and southward^32^. East Africa is predominantly semi-arid with an average rainfall amount of less than 800 mm per year; and sub-humid with an average rainfall amount varying between 800 and 1300 mm per year^26^. The vegetation of East Africa is predominantly rain-fed and agriculture has a key impact on the economy of the region, with approximately 80% of the combined population of Rwanda, Uganda, Tanzania, Burundi, Ethiopia, Kenya, Somalia and South Sudan, which depends on agriculture for their livelihood^26^. Most of the Africa’s famous mountains including Mount Kilimanjaro, Rwenzori, Virunga, Kenya and Elgon are located in East Africa^33^. The elevation of the region ranges from 0 to 5895 m above sea level (Fig. 2(a)) and bounded by 14°52′46″N and 11°43′53″S, 24°7′18″W and 51°25′1″E. The region shares its eastern border with the Indian Ocean while the north, south and western borders are surrounded by other African countries. Specifically, the Indian Ocean provides an important route for commercial activities and also serves as a moisture source for the region^34^.

## Data Sets

The Normalized Difference Vegetation Index (NDVI) has shown to be a suitable index in characterizing the continental-scale distribution and phenological seasonal dynamics of vegetation^35^. Therefore, we have used the longest available NDVI data records, from the Global Inventory Monitoring and Modeling System (GIMMS) Third Generation (3 g) Advanced Very High Resolution Radiometer (AVHRR) sensor onboard the National Oceanic and Atmospheric Administration (NOAA) satellites^4^ at a spatial resolution of 8 km and a temporal resolution of 15 days. The data sets were retrieved from https://ecocast.arc.nasa.gov/data/pub/gimms/3g.v1/ and pixels with annual averages NDVI values less than 0.1 were considered non-vegetated and excluded from analysis to minimize the influence of deserts, and sparsely vegetated areas^36^. The 15-day data was composited into monthly values using Maximum Value Composite (MVC) technique. GIMMS3g NDVI comprises of past NOAA-AVHRR records (0.083° × 0.083° resolution, 1981–2011) which has been recalibrated, and corrects for errors in trends due to calibration loss, volcanic eruptions, and orbital drift.

Climate data records used in this work were gridded observation precipitation and temperature from Climate Research Unit (CRU) of the University of East Anglia (UEA) from 1982 to 2015 at 0.5° × 0.5°. The data sets were downloaded from http://data.ceda.ac.uk/badc/cru/data/cruts/cru_ts_4.00/data/. The CRU records have been widely applied in many studies over Africa. CRU gridded observation data is arguably the most suitable climate data sets as a result of the fact that the region is data sparse. The Koppen-Geiger classification used in this work was retrieved from Köppen-Geiger classifications, following the rules given in^37^ as applied to the 5′ resolution WorldClim global climatology (www.worldclim.org; Version 1.4, release 3), were downloaded from the CliMond set of climate data products (www.climond.org).

The Digital Elevation Model (DEM) from the National Aeronautics and Space Administration (NASA) Shuttle Radar Topographic Mission (SRTM) was employed in this work. The NASA SRTM has produced and supplied DEMs for more than 80% of the users all over globe (.http://srtm.csi.cgiar.org/, approx. 90 m resolution).

The land cover map used in this work is based on Global Land Cover Map (GLCM) which are produced from MOD12C4, MOD12C5 (Boston University), GLC2000 (Joint Research Center), Globcover (European Space Agency) and UMD (The University of Maryland), GLCNMO (National Geographical Institute). The GLCM was retrieved from http://db.cger.nies.go.jp/dataset/landuse/data/regional_geotiff/Africa.tif.gz. The Extended Reconstructed Sea Surface Temperature version 4 (ERSSTv4) temperature data was used to study ENSO impact. The data records were retrieved from https://www1.ncdc.noaa.gov/pub/data/cmb/ersst/v5/. The El-nino Southern Oscillation (ENSO) signal is determined via a number of indicators from the Pacific Ocean which include the Pacific Sea Surface Temperature (SST), Southern Oscillation Index (SOI) or Outgoing long-wave radiation, OLR^28^. SOI is referred to as the sea level pressure (SLP)^38^. OLR is important for understanding changes in the Earth’s radiation energy^39^ and also used as a signal of tropical rainfall associated to ENSO related convection^28^. According to Anyamba *et al*.^29^, globally, the climate condition in the Tropics was varied by the most famous ENSO warm event of this century during the 1997/98 period and a shift to cold conditions in 1999/2000.

## Methods

### Estimation of gradual change at a constant rate in time series (model A)

In assessing the gradual changes in NDVI^40,41^, we used a linear regression model given by Eq. 1 below:

1 $${y}_{t}=a+b{x}_{t}+y\,{x}_{t}\in \{1\,\mathrm{...}\,N\}$$

where *y*_*t*_ represents the NDVI time series, *x*_*t*_ is the time span, *a* and *b* are the regression intercept and slope respectively for linear model, and *γ* is the residual of the fit. The slope (b) of the regression indicates the average temporal change and for *b* > 0, it shows increasing trends and *b* < 0, it shows decreasing trends.

### Detection of break points in time series (model B)

For a time series *x*_*t*_ = 1, 2, 3, … *N*, a Break Point (BP) is introduced into two consecutive branches of the series by a moving point k, *k* ∈ 3 + *t*, *t* = 1, 2, … *N* − 3 with a moving step i = 1 and a least-square linear regression model (Eq. 1) is fitted to each of the paired time series segments as shown in Eq. 2 ^40^:

2 $$\{\begin{array}{ll}{y}_{t}={a}_{bk}+{b}_{bk}{x}_{t}+y,({x}_{t}\le k) & k\in 3+i,\{i=1,2,\,\mathrm{...}\,N-3\}\\ {y}_{t}={a}_{ak}+{b}_{ak}{x}_{t}+y, & k\in 3+i,\{i=1,\,2,\,\mathrm{...}\,N-3\}\end{array}$$

where *y*_*t*_ is the NDVI time-series, *x*_*t*_ is the time period, k is the BP time, a and b are the intercept and trend respectively, the subscripts *bk* and *ak* are the regression constants for the two paired portions before and after *k* respectively; and *γ* is the residual of the fit. More literature about Break Points model can be obtained from Chen *et al*.^40^, from which the Akaike Information Criterion (AIC) was applied to evaluate the importance of introducing BP in the series.

3 $$AIC=n\,\log (\tfrac{R}{n})+2k+\frac{2k(k+1)}{n-k-1}$$

where *R* is the residual sum of squares, *n* is the sample size, and k is the number of parameters in the model in the estimated model. A BP was introduced if the AIC of the piecewise regression was smaller than the ordinary least square regression.

### ENSO impact composite images

In this work, El Niño, neutral, and La Niña years were chosen based on data from sea surface temperature (SST) anomalies of the tropical Indian Ocean in the region +0.5 °C and −0.5 °C referred to as the Niño 3.4 region. We considered the gridded Extended Reconstructed Sea Surface Temperature version 4 (ERSSTv4) temperature data^42^. For average SST anomalies exceeding +0.5 °C (−0.5 °C) from October to March, the preceding growing season belonged to an El Niño (La Niña) year. The October to March period typically coincides with peak ENSO Conditions Neutral years ranged between −0.5 °C <SST < +0.5 °C as shown in Table 1 ^43^. NDVI composites were computed for all years selected as El Niño, La Niña and neutral. The deviation and sum of each variable was computed in each season to identify variations in croplands over East Africa.

**Table 1:** Extraction of El Niño, Neutral, and La Niña years between from 1982 to 2015 using ERSSTv4 SST Anomalies.

El nino years (13)	Neutral years (8)	La nina years (13)
1987–88	1982–83	1981–82
1997–98	1988–89	1983–84
2001–02	1990–91	1984–85
2002–03	1998–99	1985–86
2003–04	1999–00	1986–87
2006–07	2000–01	1989–90
2008–09	2004–05	1991–92
2009–10	2007–08	1992–93
2010–11		1993–94
2011–12		1994–95
2012–13		1995–96
2013–14		1996–97
2014–15		2005–06

### Extraction of phenological changes

The start and end dates of a season are important variables which are usually chosen as a measure of phenological shifts. Several techniques have been developed to study vegetation phenology (SOS, POS, EOS and LOS) from satellite data. For example, Bobée *et al*.^5^ as well as Gong *et al*.^44^ used mid-point value where the empirical equations was adopted and inflection points used. In this work, the Start of season (SOS), End of season (EOS) and Peak of Season (POS) will be extracted using the inflection method developed by Gong *et al*.^44^. In this method the SOS is obtained by the highest turning point (local maxima) of the second derivative while the EOS is obtained by the lowest turning point of the local (minima) using second derivative and POS represents the turning point of the global minimum using second derivative.

### Standardize anomaly

Anomalies were computed to show a clear understanding between vegetation dynamics and climate variability. The monthly and seasonal standardized anomalies (*std*. Anomaly) for vegetation and climate parameters were computed using the equation given below

4 $$std.{\rm{Anomaly}}=\frac{{X}_{i}-\overline{\overline{X}}}{\delta }$$

where *X*_*i*_ is the value of NDVI/climate at a particular time (month/season), $\overline{\overline{X}}$ and *δ* are the average (monthly/seasonal) and standard deviation (monthly/seasonal), respectively, over the study time period, 1982–2015^45^. Monthly and seasonal standardized anomalies of all the parameters were computed to carry out the correlation analysis between NDVI and climatic parameters. Temporal trends were also computed on those anomaly time series.

The Anomaly time-series of all the variables were used for correlation analysis (*r*_*xy*_) as this helps in assessing if climate extremes results in extreme vegetation activity. The result shows where vegetation may react sensitively to climatic variability which is important in understanding climate change.

5 $${r}_{xy}=\frac{{\sum }_{i}({x}_{i}-\bar{x})({y}_{i}-\bar{y})}{\sqrt{{\sum }_{i}{({x}_{i}-\bar{x})}^{2}{\sum }_{i}{({y}_{i}-\bar{y})}^{2}}}$$

And t-test was done according to

$$t=\frac{{r}_{xy}\cdot \sqrt{n-2}}{\sqrt{1-{r}_{xy}}}$$

Where y and x are the predictor (climate) and response (NDVI) variables, respectively. Cross-correlation coefficient was also used to study the Lead/lags in vegetation and climate parameters

### Detrended fluctuation analysis (Hurst scaling exponent index)

In recent years the Detrended Fluctuation Analysis (DFA) method is widely used for the determination of Hurst exponent in several fields including Geosciences and climate timeseries^46^ (Kantelhardt *et al*., 2002). A Hurst exponent of H < 0.5 (>0.5) shows an anti-persistent (persistent) process, which implies that the increment of a data signal are likely to be followed by decreases (increments) over time. For random signals, H = 0.5 which implies that there is no persistence and the signal resembles a Brownian motion^47^ (Dai, 2016). The steps^46,48^ (Kolscienlny Bunde, 2006, Igbawua *et al*., 2019) are given below

- The initial NDVI time series B with length *N*, is integrated and subsequently detrended using
  $$y(k)=\mathop{\sum }\limits_{t=1}^{N}[B(i)-\bar{B}]$$
  where $\bar{B}$ is the mean
- The detrended series of NDVI is then divided into equal length n, to each segment a polynomial fit is computed, the resulting vector *y*_*n*_(*k*) is the subtracted from *y*(*k*)
  6 $$F(k)=\sqrt{\frac{1}{N}\mathop{\sum }\limits_{k=1}^{N}{[Y(k)-{y}_{n}(k)]}^{2}}$$
- This procedure is applied to each segment.
- The graph of logarithmic fit of *F(k)* versus is produced and the slope of the fit gives the Hurst exponent.

### Coefficient of variation

The mean coefficient of variation (CV) of each of the 12 000 spatially distributed NDVI values for a single month (e.g., January) over the 34-year period and doing same for each of the 12 months. The main aim for determining the coefficient of variation (CV) was to study the region’s NDVI responses to the large variations in climate (i.e., temperature and precipitation). With the reference made from Barbosa *et al*.^49^, CV is given as in Eq. 7

7 $$CV=\frac{\delta }{\bar{x}}$$

### Normalized seasonal cycle (NSC)

The method of estimating the NSC, adapted from Igbawua *et al*.^41^, was done to study the possible correlations and lags. For this research, the monthly (*m*_*av*_) and yearly averages (*y*_*av*_) of NDVI and climate parameters were normalized as given in Eq. 8. The normalized values were later plotted against the number of months to study the lags and possible correlations.

8 $$NSC=\frac{{m}_{av}}{{y}_{av}}$$

## Results and Discussions

### The 34-year mean climate and NDVI over east africa

**Figure 3:**
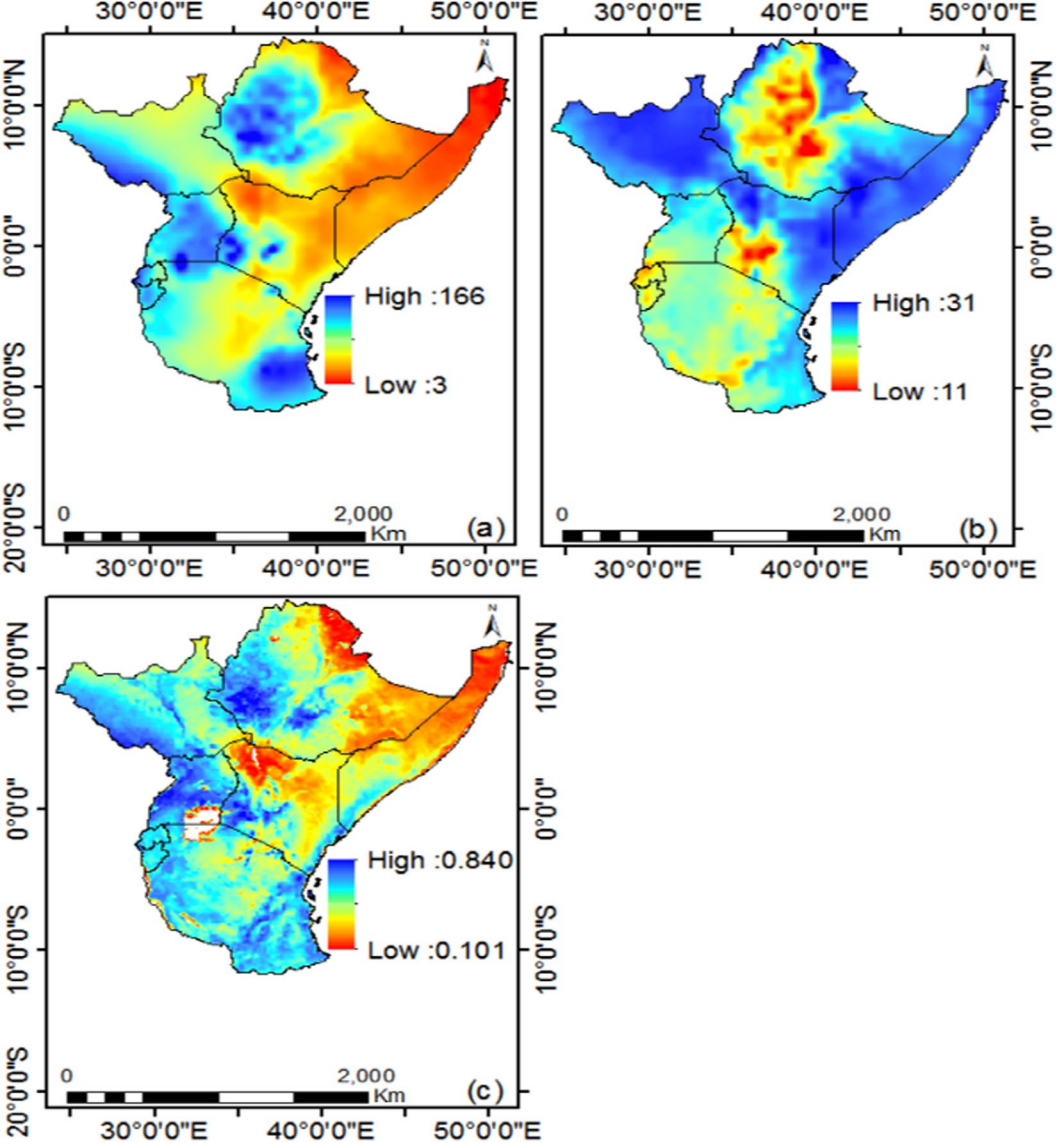
Mean (**a**) Precipitation (mm/month), (**b**) Temperature (°C) and (**c)** NDVI from 1982 to 2015 over East Africa.

The spatial distribution of the 34-year mean monthly precipitation, temperature and NDVI over East Africa are presented in Fig. 3. Results indicates that high values of precipitation corresponded with low values temperature, especially north east of the study region. The spatial distribution of mean NDVI agrees well with precipitation than temperature. Non-vegetated areas over East Africa accounts for about 1.1% of the total study area, while vegetated areas with NDVI in the range 0.1–0.4, 0.4–0.5, 0.5–0.6 and 0.6–0.8 accounts for 33.0, 23.6, 26.5 and 15.8% of the study area with evident spatial heterogeneities. The major vegetation cover types surrounding the region are grasslands, woodlands and agricultural lands (croplands) (Fig. 2(b)) with NDVI values in the range of 0.2–0.6. High NDVI values of >0.7 are mostly located in the forest areas, in the northwest and around the great lakes and Mount Kilimanjaro. The mean NDVI of croplands at grid point is about 0.629 which is higher than the mean regional NDVI (0.53) at grid point. This shows that any decrease (increase) of cropland vegetation might affect the regional NDVI of East Africa.

Table 2 shows summary of result computed by using long term averages of NDVI, precipitation and temperature from 1982 to 2015 for El nino, Neutral and La nina years over all seasons. Result shows that El Niño conditions are associated with an overall increase in surface temperatures in all the seasons, with the highest value of +0.48 °C in the MAM season, corresponding to low percentage increases from mean value for both NDVI and precipitation. Also, La nina conditions are associated with an overall decrease in surface temperatures in all the seasons with the lowest value of −0.24 °C in SON season and high percentage increase of NDVI and precipitation MAM season. Basically, the climate of East Africa is influenced by ENSO events, which cause severe extreme conditions, tend to enhance precipitation over the region except in the northern and western parts of the Ethiopian and Eritrean highlands, where drought and floods are prevalent.

**Table 2:** Summary of result computed by using long term averages of NDVI, precipitation and temperature from 1982 to 2015 for El nino, Neutral and La nina years over all seasons.

	Season	DJF	MAM	JJA	SON
El nino	NDVI (%)	38.4	37.9	38.5	38.2
	Precipitation (%)	42.0	37.0	39.3	39.8
	Temperature (Diff. from mean)	+0.45	+0.48	+0.33	+0.33
Neutral	NDVI (%)	24.0	23.6	23.4	23.4
	Precipitation (%)	22.0	22.0	23.9	22.9
	Temperature (Diff. from mean)	−0.04	−0.09	0.0	−0.09
La nina	NDVI (%)	37.6	38.5	38.1	38.3
	Precipitation (%)	36.0	41.0	36.8	37.4
	Temperature (Diff. from mean)	−0.41	−0.39	−0.33	−0.24

### Temporal NDVI dynamics over the region

**Figure 4:**
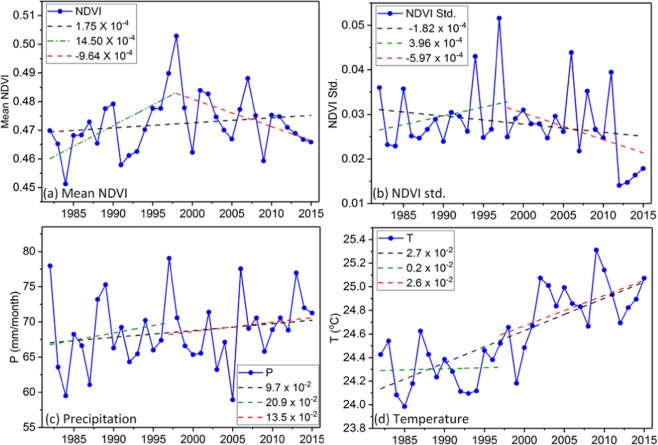
Interannual variation of (**a**) mean NDVI, (**b**) NDVI standard deviation, (**c**) precipitation and (**d**) temperature from 1982 to 2015.

Figure 4(a,b) show the interannual variation of annual mean NDVI and NDVI standard deviation over the East Africa from 1982 to 2015. The East Africa region indicated an increasing trend which is evidence of favorable agricultural and land use practices in recent decades. The linear regression model (model A) in Eq. 1 was used to obtain a statistically insignificant and positive (negative) linear trend of 1.75 × 10^−4^/yr (−1.82 × 10^−4^) for NDVI (NDVI std. dev) at p > 0.05. The BP was detected by using the piecewise regression model (model B) in Eq. 2 based on the 34-yr data records as 1998. Results show that before the NDVI Break Point (BP), the mean annual NDVI (NDVI std. dev.) increased at 14.5 × 10^−4^/yr (3.96 × 10^−4^) and decreased insignificantly for NDVI (NDVI std. dev.) at −9.64 × 10^−4^/yr (−5.97 × 10^−4^) after the BP with p > 0.05. To test the significance of model A and B, we applied the Akaike Information Criterion (AIC) (Eq. 3). The AIC of model A and B is −208.9 and −201.4 respectively for mean NDVI, while the AIC for model A and B is −223.9 and −225.2 respectively for NDVI std. dev. Models with smaller AICs are selected over those with larger ones. This shows the linear regression model A and piece wise regression model B are robust for detecting gradual and break points in mean NDVI and NDVI standard deviation respectively. From Fig. 4(a,b), the negative trends observed in NDVI standard deviation after the BP is as a result of the improved vegetation greening from below normal conditions from 1980s and 1990s to above normal conditions in 2000s and 2010s. Both, precipitation and temperature have shown insignificant positive trends (p > 0.05) of 9.7 × 10^−2^/yr and 2.7 × 10^−2^/yr, respectively, over the study region from 1982 to 2015 (Fig. 4(c,d)). Since the regression model (i.e. model A) shows significance in AIC, the regression slope of NDVI over croplands was done, and results reveal that the vegetation of the croplands (non-croplands) over East Africa changed insignificantly 6.9 × 10^−5^/yr (5.16 × 10^−4^/yr). This conveys that non-croplands are fast getting reduced. This in turn, affects the entire vegetation cover of the entire region, eventually, resulting to cropland degradation (See Appendix I). Also, the NDVI of cropland was at an all time low (from 1982 to 2015) of 0.607 in 2012. We also conducted same analysis across the individual months from 1982 to 2015 using mean NDVI to assess NDVI changes at smaller scales. The annual or growing season NDVI trends are normally obtained as the average of trends in each month^40^ (Table 3).

**Table 3:** Statistical parameters of model A and model B using monthly mean NDVI time series from 1982 to 2015 over East Africa.

Month	Mean	Trend			AIC (A)	AIC (B)
		Overall (A)	Before BP	After BP		
Jan	0.455	1.60E-04	20.5E-03	−1.86E-03	−144.4**	−135.1
Feb	0.438	5.98E-04	19.7E-03	−7.00E-04	−155.5**	−145.4
Mar	0.438	5.28E-04	16.7E-04	6.54E-04	−161.6**	−154.9
Apr	0.473	−0.45E-04	16.9E-04	−1.12E-03	−167.8**	−165.9
May	0.516	−5.17E-04	17.0E-04	−2.53E-03	−170.3**	−170.0
Jun	0.493	−0.05E-04	15.1E-04	−1.36E-03	−177.1**	−173.9
Jul	0.469	−2.00E-04	14.8E-04	−1.44E-03	−182.9	−183.4**
Aug	0.467	0.39E-04	15.7E-04	−1.35E-03	−181.1	−191.4**
Sep	0.470	0.64E-04	10.1E-04	−1.05E-03	−186.9**	−186.0
Oct	0.474	5.22E-04	5.3E-04	3.41E-04	−171.1	−173.4**
Nov	0.488	6.62E-04	8.9E-04	−4.07E-04	−155.1	−158.1**
Dec	0.486	3.38E-04	12.7E-04	−7.40E-04	−141.4	−141.9**

** indicates significance in selecting a model based on AIC.

For model B, all the months showed positive significant trends before BP and negative trends except Mar and Oct. The detected BPs shows variation from month to month. However, the AIC of model B in Jul, Aug, Oct, Nov and Dec are smaller than AIC of model A, which shows that model B is the most preferred model in modeling vegetation dynamics in those months from 1982 to 2015. In this study, the mean NDVI initially continued to increase until 1997/98, before it showed a decreasing trend until mid-2000s. This could be due to drying, as result of reduced precipitation and increase of temperature of temperature. Even thought, there is a drop in the mid-2000s after break point, the overall trend in NDVI remains positive same as precipitation. Also, there is an observable peak in precipitation in 1997/98 same year that the peak occurred in NDVI which corresponds with one of the deadliest ENSO episodes over East Africa.

### NDVI-ENSO relationships

The main moisture source of East Africa is from the Indian Ocean through the southeasterly trade winds with long (short) rains during the MAM (SON). The Intercontinental Convergence Zone (ITCZ) has a biannual convergence with seasonal reversal of the trade winds. The recession in the Indian Monsoon system over East Africa is responsible for placing constraints on convection, and the limitation to southerly flow arising from the Ethiopian highlands, resulting in the southwesterly flow of moisture. Most flood and drought occurrences over the region are finger prints of ENSO impacts and documented reports have shown that peak periods of ENSO are from October to March. This period coincides with the SON and MAM seasons and increased rainfall in East Africa.

**Figure 5:**
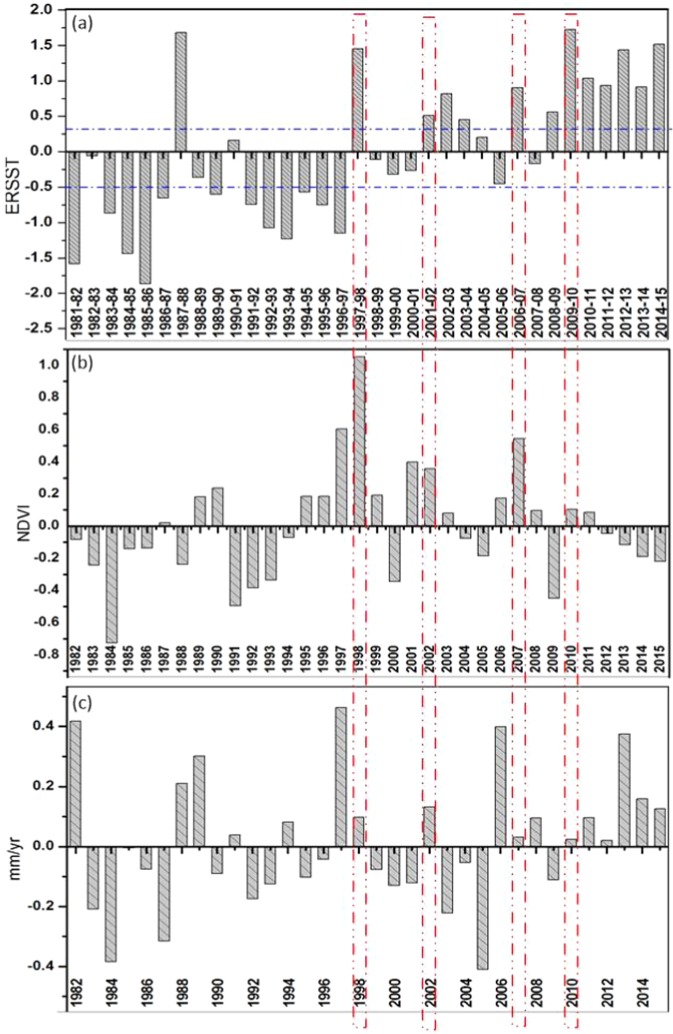
Annual (**a**) ERSST, (**b**) NDVI, (**c**) Precipitation anomalies from 1982 to 2015.

From Fig. 5, not all ENSO events over the region matched with the effects observed over East Africa, arguably most of the multi-year ENSO events were captured. In those years that corresponded with Neutral, El Nino, and La Nina events, the reconstructed data and episodes rhymed with the expected ENSO effects. Also, it was observed in the results that the data could not capture the details about vegetation and climate dynamics. According to Glennie & Anyamba^50^, several reasons are behind the discrepancies between ENSO events and impacts on croplands. They include abnormal weather conditions which are not necessarily associated to ENSO episodes but distorts the values in the composite images, improved response of individuals to ENSO events and general resilience of crops which could have relieved such effects from temperature and precipitation.

The major flood and drought episodes over the East Africa have been linked to ENSO events. For the 1997/98 event, high SST resulted in high positive anomalies for both NDVI and precipitation while in 2014/15 event, precipitation (NDVI) recorded positive (negative) anomalies. Specifically, the ENSO effect may result in either drought conditions or rain induced cropland damage due to flood. In this work, Pearson correlation of NDVI of croplands from 1982 to 2015 is done in Neutral, El Nino, and La Nina years to study the cropland changes over time (Table 4).

**Table 4:** Pearson correlation between NDVI versus temperature and precipitation from 1982 to 2015 across the seasons.

Correlation between	DJF	MAM	JJA	SON
**El nino years**				
NDVI vs Precipitation	0.728 (0.005)*	0.284 (0.347)	−0.038 (0.903)	−0.123 (0.687)
NDVI vs Temperature	0.099 (0.746)	0.053 (0.865)	−0.147 (0.632)	−0.077 (0.802)
Precipitation Vs Temp.	0.000 (0.999)	−0.129 (0.674)	−0.645 (0.017)*	0.126 (0.683)
**Neutral years**				
NDVI vs Precipitation	0.121 (0.775)	0.023 (0.957)	−0.294 (0.479)	0.719 (0.044)*
NDVI vs Temperature	0.571 (0.134)	−0.143 (0.735)	0.221 (0.560)	0.581 (0.131)
Precipitation Vs Temp.	−0.157 (0.710)	−0.755 (0.030)*	−0.224 (0.593)	0.042 (0.922)
**La nina years**				
NDVI vs Precipitation	0.605 (0.028)*	0.851 (0.000)*	0.026 (0.933)	0.616 (0.025)*
NDVI vs Temperature	−0.005 (0.987)	0.028 (0.924)	0.407 (0.167)	0.270 (0.372)
Precipitation Vs Temp.	−0.172 (0.572)	−0.237 (0.436)	−0.093 (0.764)	0.452 (0.121)

^*^shows significant correlation p < 0.05.

Figure 5(a,b) shows annual (a) ERSST, (b) NDVI, (c) Precipitation anomalies from 1982 to 2015.

It can be noted that there exist some tele-connections between NDVI and ENSO during most of the El-nino and La-nina episodes, specifically 1992/93 and 1997/98. The pattern of anomalies exhibited by vegetation agrees well with precipitation. In Fig. 5(a,b), the 1997/98 El-nino year corresponded with high NDVI and rainfall values and decreased NDVI and rainfall values in 92/93 indicated slight negative anomalies in the SST. The NDVI values during the 1997 (1998) are 1.3% (3.5%) above normal and 1.4% (0.4%) below normal (slightly above normal) in 1992 (1993). The above-normal NDVI was a result of excessive precipitation in 1997 which spilled up into 1998. Specifically, the damage caused by ENSO to vegetation is transferred via precipitation^4^. The detected weak link between mean NDVI and ENSO suggests that there may be a weak teleconnection between NDVI and ENSO. The effect of ENSO on NDVI series is strongest when considering NDVI in seasons prior to the actual months, suggesting a time lag between them. From Fig. 5(d), the strongest effect was observed between OND SST and December NDVI. A critical assessment shows that the build-up to this effect starts from SON short rainy season before spilling over to long rainy season.

### Persistence of NDVI trends over East Africa

**Figure 6:**
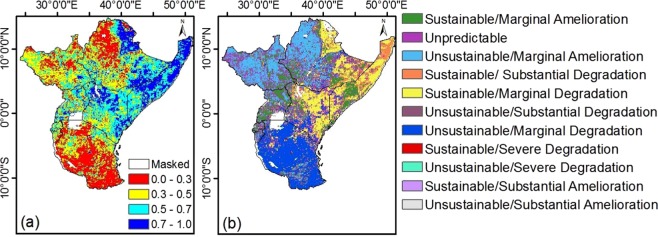
(**a**) Spatial distribution of Hurst exponent and (**b**) sustainability of the mean NDVI over East Africa from 1982 to 2015. The spatial images of NDVI slope and the Hurst exponent have been superimposed in (**b**).

**Figure 7:**
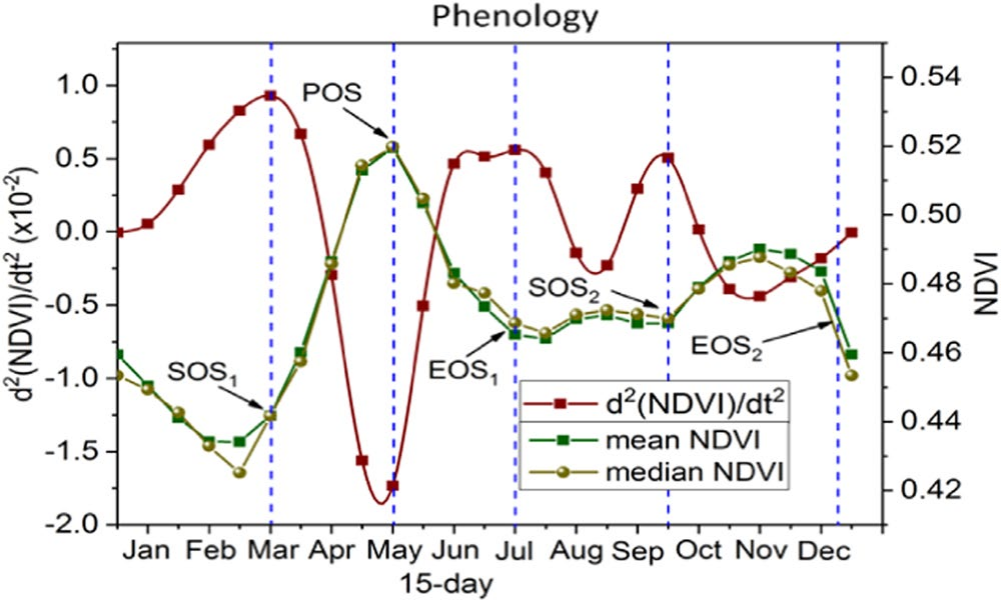
Phenology Metrics over the study region. (SOS: start of growing season; POS: peak of growing season; EOS: end of growing season, LOS: Length of growing season).

Figure 6, shows a feasible approach in representing and mapping NDVI sustainable over East Africa. The approach involves superimposing the NDVI slope map and the Hurst exponent^35^ to produce a vegetation growth change zones. This comprise of the direct and indirect changes which are very difficult to be captured by ordinary regression model but can better be seen where superimposed with the scaling power law Hurst exponent. In Fig. 7a, vegetated (non-vegetated) areas with high (low) vegetation cover may have shown high values of Hurst exponent >0.5 as a result of the fact that high (low) values of NDVI did followed high (low) NDVI values. This means NDVI in such areas has persisted under different conditions. For vegetated areas with Hurst = 0.5, high (low) values of NDVI follow low (high) values as a result of rapid changes arising from vegetation degradation and recovery. We have presented the statistical analysis **(**Table 5) of change prognosis which have aided the classification of the sustainability classes and persistency of vegetation trends over East Africa. In this research work, about 18.63% pixels exhibited a behavior, typical of random walk (H = 0.5). This suggest that the NDVI growth changes may eventually persist, overturn or fluctuate randomly in the future depending of the drivers. Generally, 12% pixels in the study area indicated Sustainable/Amelioration over countries like Rwanda, Uganda, Burundi, Central part of Somalia, Ethiopia, Southern part of Kenya and South Sudan. More so, are located along the shores of Somalia, Northern part of Ethiopia and close to the ocean. Also, about 25% pixels covered areas with Unsustainable/Degradation mostly located in Burundi, Tanzania and southern part of Kenya. For Unsustainable/Amelioration, the area represented about 20% of the total study area covering countries like Kenya, Somalia and Ethiopia. However, 24% pixels indicated Sustainable trends, the prediction is that, they will continue to degrade over time. In Table 5, the mean climate elements from 1982 to 2015 over the vegetation change type has been presented. Results reveal that precipitation over the sustainable areas is >68 mm/month with favorable temperatures. Arguable, some areas with unsustainable trends also shows favorable precipitation and temperature. This suggests that climate may not be the only drivers of vegetation change over East Africa. This corroborates with Ndayisaba *et al*.^35^ who did a similar work but using rescale range approach over Rwanda to compute Hurst exponent.

**Table 5:** Comparison between NDVI change trend, the Hurst exponent and mean climate elements from 1982 to 2015.

Temperature °C/month	Precipitation Mm/month	NDVI range	Hurst exponent	Vegetation Change type	%
24.8	69	0.0004–0.03	>0.05	Sustainable/Marginal Amelioration	12.12
24.6	72	0.0004–0.03	=0.05	Unpredictable (Brownian motion)	18.63
24.2	88	0.0004–0.03	<0.5	Unsustainable/Marginal Amelioration	20.25
26.6	33	−0.0004–0.0004	>0.05	Sustainable/Substantial Degradation	7.43
26.3	40	−0.0006–(−0.0004)	>0.05	Sustainable/Marginal Degradation	16.82
22.6	84	−0.0004–0.0004	<0.5	Unsustainable/Substantial Degradation	5.04
22.9	77	−0.0006–(−0.0004)	<0.5	Unsustainable/Marginal Degradation	19.46
22.7	61	≤−0.006	>0.05	Sustainable/Severe Degradation	0.08
21.6	63	≤−0.006	<0.5	Unsustainable/Severe Degradation	0.02
25.0	95	≥0.03	>0.05	Sustainable/Substantial Amelioration	0.15
19.9	102	≥0.03	<0.5	Unsustainable/Substantial Amelioration	0

### NDVI phenology metrics over east africa from 1982 to 2015

The results of the average phenology dates (SOS, POS, EOS, LOS) at over the study area was determined using GIMMS 3 g NDVI from 1982 to 2011 (Fig. 7).

Overall, there exist two start of growing seasons (SOS) in response to the two rainy seasons: MAM and SON. The first growing season start date SOS_1_ was obtained as the local maxima of the 2^nd^ derivative of NDVI with time during the start of rainy season while the peak of season (POS) is the local minima after the SOS. The end of the first growing (EOS_1_) is obtained as local maxima after the peak of growing season (POS). The second growing season (SOS_2_) dates are extracted in the same way. From Fig. 7, all the events that describes SOS_1_, EOS_1_, SOS_2_, EOS_2_, and POS have rhymed with the sequence of mean and median NDVI. In both seasons, there is a time lag of 1–2 to months between NDVI and precipitation. Thus the LOS during the first (second) rainy season is from March to July (September to December). The peak of season occurred in May with mean NDVI of 0.52. The results of this analysis also suggest that vegetation phenology is significantly related to rainfall over the study region, where the vegetation types are generally grassland, croplands, savannas and shrublands. The spatial distribution of vegetation is partly associated with soil properties and the amount of rainfall received in the region. There is sufficient moisture in the soil to trigger vegetation growth during SOS. However, areas with negative correlations between NDVI and precipitation during SOS suggest that the reductions in temperature and solar radiation caused by excessive precipitation may be unfavorable for vegetation growth^24^.

### Spatio-temporal relationship between NDVI and climate parameters

**Figure 8:**
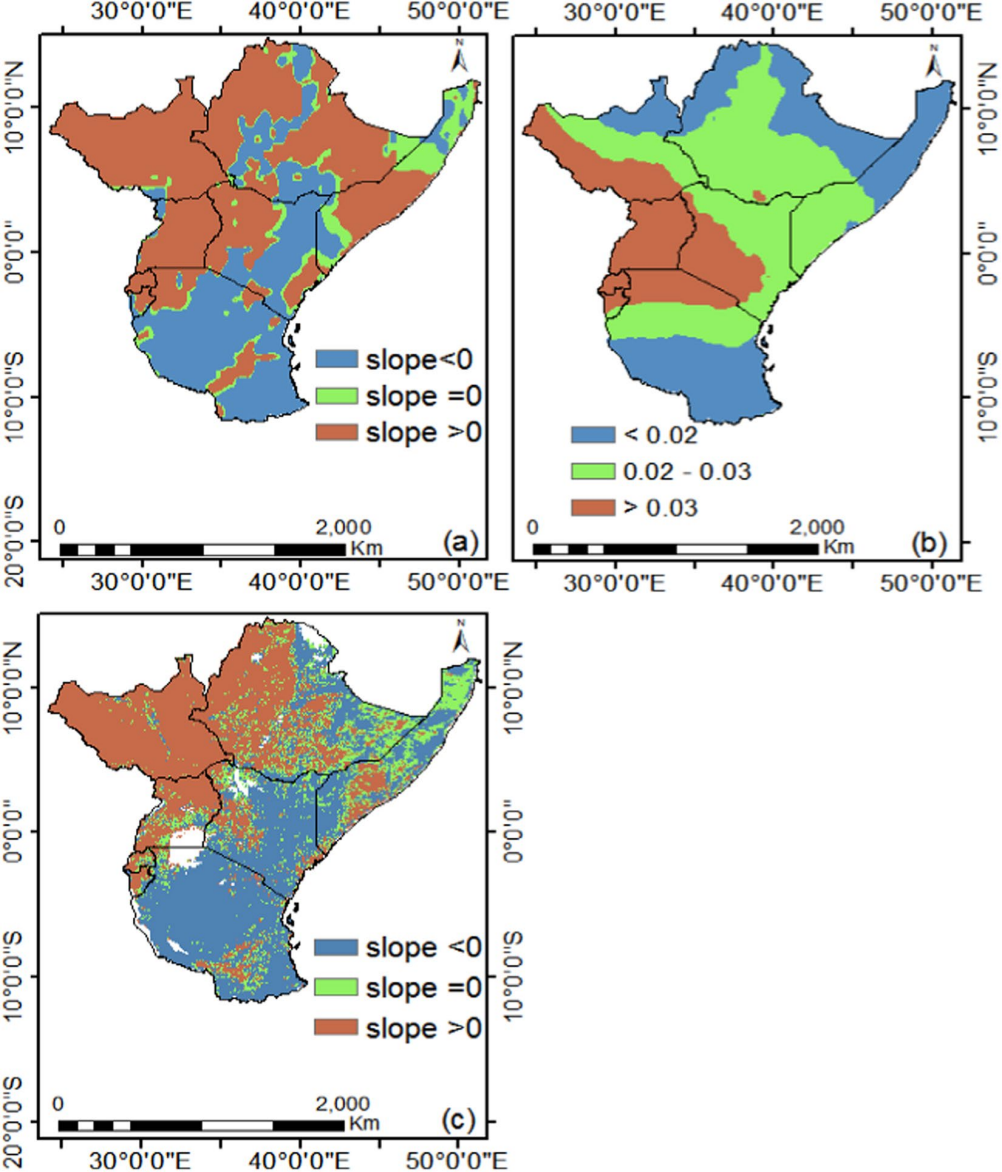
Spatial distribution of (**a**) precipitation, (**b**) temperature and (**c**) NDVI trends from 1982 to 2015.

The CRU mean annual precipitation for the study area from 1982–2015 ranged from 3 to 166 mm/month, and approximately 31.9 and 24.5% of the precipitation occurs during the MAM long and SON short rainy season, respectively. This results show that the MAM rainy season is wetter than the SON season. Generally, there is a change in shift of the NDVI after 1998, which indicates that the NDVI slightly decreased with precipitation slightly increasing and temperature rising (see Fig. 5). Figure 8 shows the spatio-temporal relationship between NDVI and climate parameters. From Fig. 8(a), the spatial distribution of trends in precipitation indicates that, about 60.6 and 39.4% pixels are increasing and decreasing respectively while less significant amount remain unchanged. In Fig. 8(b), the temperature indicated that about 99% pixels increased while about 1% pixels remained unchanged and no pixel showed negative trend. For NDVI, about 47.0 and 49.1% pixels are increasing and decreasing, respectively, while less significant amount remain unchanged from 1982 to 2015 (Fig. 8(c)). The long-term decrease of vegetation as indicated by NDVI is a sign of degradation^51^. For instance, the spatial trend of precipitation showed high increase in long term precipitation and the spatial distribution of NDVI decreased over time in most areas across the study region. This inverse response suggests that, there could be vegetation degradation as a result of deforestation and urbanization. Alternatively, areas with decreasing trend of precipitation with respect to decreasing; there is an indication of climate induced vegetation changes which are discernible in the Southern part of study area (Fig. 8(a,c)).

**Figure 9:**
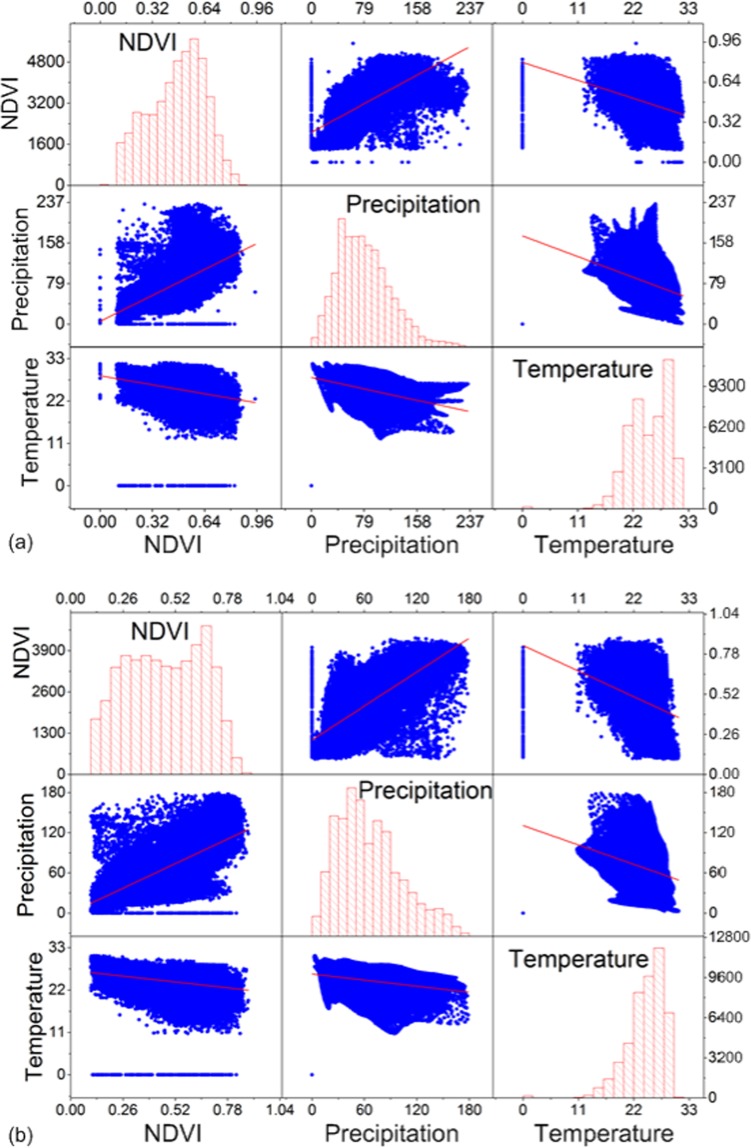
Matrix plot of NDVI and climate parameters for (**a**) long rainy (MAM) and (**b**) short rainy season (SON) from 1982 to 2015.

In Fig. 9, NDVI showed positive (negative) relationship with precipitation (temperature) both in the long and short rainy seasons. Also, all the two seasons, temperature is inversely related with precipitation. The diagonal variables in Fig. 9 are the histograms of pixel distribution in NDVI, precipitation and temperature. NDVI pixels in long and short rainy seasons are unimodal and bimodal, respectively. Temperature is left skewed in all the seasons and precipitation is right skewed. The same relationship between NDVI and climate parameters is observed using spatial data records.

**Figure 10:**
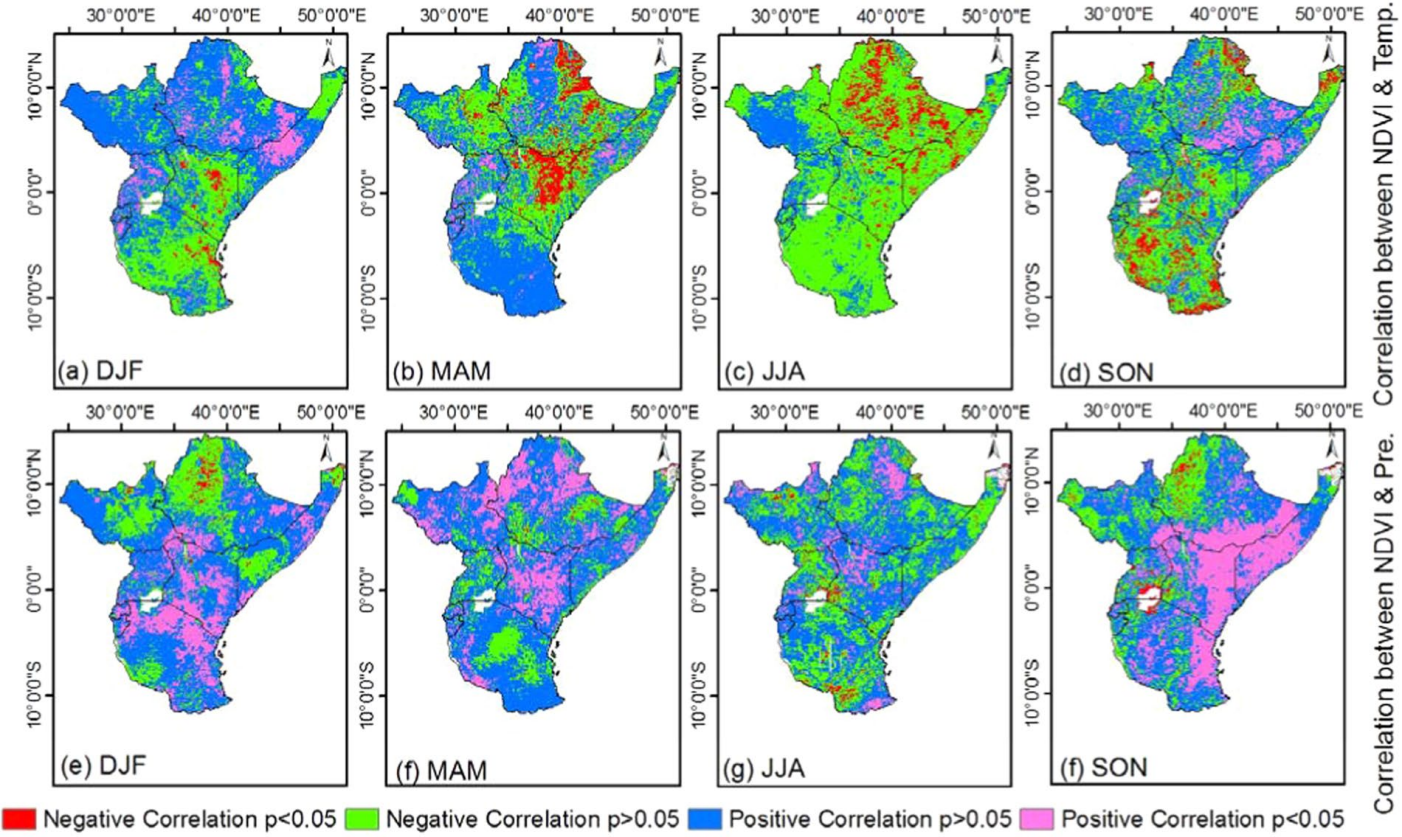
Correlation between NDVI and precipitation for (**a**) long season (MAM) and (**b**) short season (SON) from 1982 to 2015. (**c**,**d**) are correlation between NDVI and temperature for long and short season, respectively.

We used Pearson correlation to study the relationship between vegetation growth changes and climatic elements over the region from 1982–2015 (Fig. 10). Generally, NDVI significantly correlated precipitation than temperature. Thus, precipitation influenced vegetation growth changes than temperature. On one hand, correlation analysis of individual seasons across the region shows that 20, 23, 10 and 34% NDVI pixels positively and significantly correlated with precipitation over the DJF, MAM, JJA and SON seasons respectively while about 2, 1, 2 and 2% NDVI pixels negatively and significantly correlated with precipitation over the DJF, MAM, JJA and SON seasons respectively. On the other hand, correlation analysis of individual seasons across the region shows that 10, 5, 0 and 9% NDVI pixels positively and significantly correlated with temperature over the DJF, MAM, JJA and SON seasons respectively while about 2, 11, 10 and 9% NDVI pixels negatively and significantly correlated with precipitation over the DJF, MAM, JJA and SON seasons respectively. Thus, the DJF MAM and SON seasons are strongly driven by precipitation variation.

Most forest regions indicated low NDVI-precipitation correlations but high NDVI-temperature correlations over East Africa. This observation is consistent with the studies by Ndayisaba *et al*.^35^ over East Africa and Igbawua *et al*.^41^ over West Africa. Over the forested areas, the water budget for NDVI is high throughout year which is while NDVI reacts poorly to precipitation. In this case, temperature becomes the limiting parameter over the forested areas. Conversely, precipitation is the limiting parameter in areas with low moisture budget such as the savannas and agricultural lands. The spatial analysis of correlation between NDVI and precipitation has indicated that mean growing season NDVI is better correlated with precipitation in low plains of Eastern region than in high altitude during the short season. Figure 10 revealed that the two rainy seasons have a different mechanism by which NDVI responds to climate parameters. NDVI correlates differently with precipitation/temperature during the long and short rainy seasons. This suggests that the moisture source in each of the season has an influence on the vegetation dynamics over East Africa.

**Figure 11:**
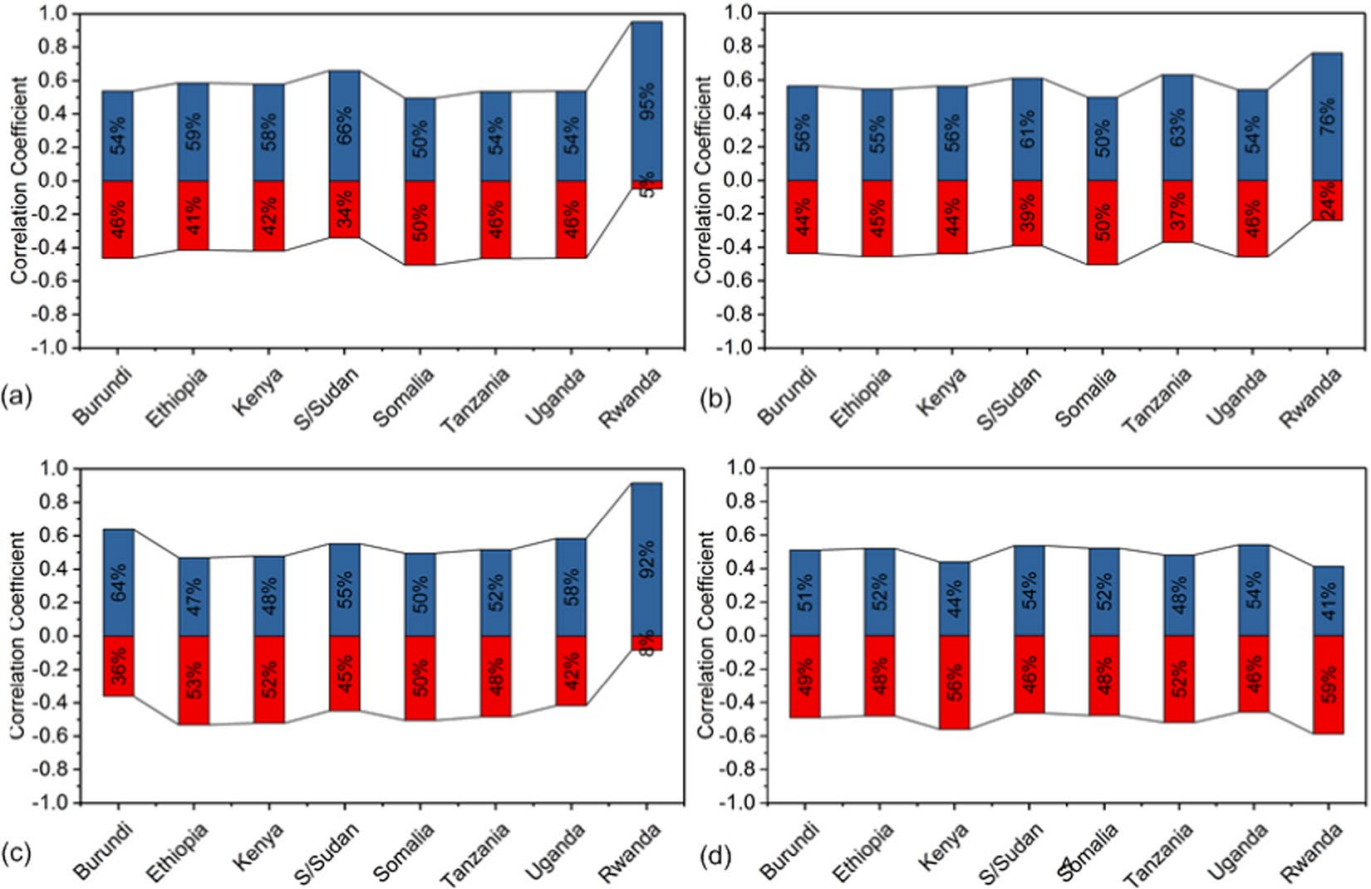
Correlation between NDVI and precipitation at country scale for (**a**) long season (MAM) and (**b**) short season (SON) from 1982 to 2015. (**c**,**d**) are correlation between NDVI and temperature at country scale for long and short season, respectively.

At country scale, the correlation coefficient of NDVI versus precipitation showed that positively correlated pixels were ≥50% of the total vegetated area for both the long and short rainy seasons (Fig. 11(a,b)). Also, during the long rainy season the correlation coefficient of NDVI versus temperature showed that all countries in study area except Ethiopia and Kenya had positively correlated pixels ≥50% out of the total vegetated area. These areas are primarily located in the northern and central part of the study area. For the short rainy season, the correlation coefficient of NDVI versus temperature revealed that all the countries in study area except Kenya, Tanzania and Rwanda had positively correlated pixels ≥50% of the total vegetated from 1982 to 2015 (Fig. 11(c,d)). The research findings have illustrated just like Ndayisaba *et al*.^35^, that the region which is basically a cool and dry equatorial climate regime (Fig. 2c) has precipitation as the limiting factor for vegetation development. Thus the sensitivity of vegetation to climate change is aligned to precipitation changes more than temperature changes. On the whole, temperature increases may exacerbate surface evaporation and subdue vegetation growth as a result of excessive water loss^46^.

### Seasonal cycle of NDVI and climate elements

**Figure 12:**
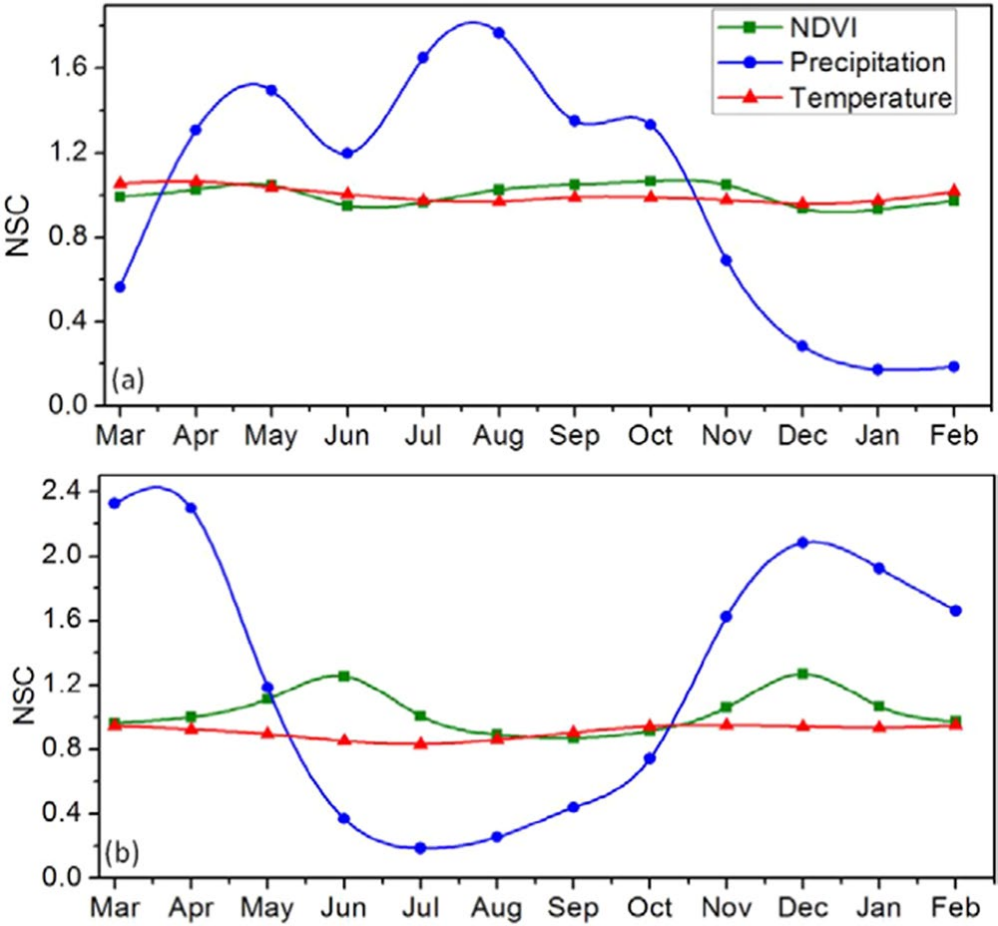
Normalized Seasonal Cycle of NDVI and Climate Elements over (**a**) North equatorial and (**b**) South equatorial regions of East Africa.

By means of the results for NSC of NDVI and climate data records, the fluctuations in the timing of the seasons and phase changes with respect to the normal annual mean have been quantified from 1982 to 2015 (Fig. 12). On average, rainfall has a higher value for time variation, and followed by NDVI, then temperature. In East Africa, the response of vegetation naturally comes after the seasonal progression of the rainfall^29^. Figure 12 illustrates Normalized Seasonal Cycle (NSC) of NDVI and climate elements, the NSC was computed using Eq. 7. The seasonal mean NDVI and climate factors computed from 1982 to 2015 have shown to exhibit seasonality pattern. Seasonality is more pronounced over the south equatorial region with steady increase during the two growing season time slope. Generally, NDVI shows high values over the south equatorial region than north equatorial region both in the MJJ and NDJ seasons. It can be observed that NDVI shows similar phase changes in the Northern equatorial (NE) region than the Southern equatorial (SE) region of East Africa. In both regions, precipitation shows high variability than vegetation and temperature.

In the NE region, NDVI increases slightly from March, peaked in May decreases in June before increasing again from August to December while in SE region, NDVI increases greatly from March to August, then decreases in September before increasing again from October to February. Over the NE region, low rainfall and moderate NDVI values are found during SOS in MAM season, and they increase with advancement of season while temperature shows slight variation. Both NDVI and temperature shows slight variation from above normal to below normal conditions. In OND season, the changes in temperature are not obvious while NDVI and rainfall shows an abrupt decrease. Similarly, over the SE region, high rainfall and low NDVI values are found during SOS in MAM seasons and as rainfall decreases, NDVI increases with advancement of season. The temperature shows slight decrease in MAM season. In OND season all the variables increased except temperature. NDVI and temperature shows lags of between 1–2 months during the JJA and OND seasons over NE and SE regions. This is because during dry-down, decreasing soil moisture conditions corresponds with increasing surface temperatures and this explains the reason for inverse relationship between NDVI and temperature over the study region. Specifically, East Africa is predicted to experience increased precipitation along with climate variability but surface runoff which is affected by precipitation and temperatures may wane due to increased temperatures^7^.

Also, the NDVI seasonal cycle is partially in-phase with climate variables over NE in MAM and OND seasons. Over the SE, NDVI is out-of-phase with climate variables only in MAM season with lags between 1–2 months. On close inspection in both regions the striking similarity between NDVI and rainfall becomes visible, maximum NDVI values are reached after the rainfall season begins to subside and NDVI is totally out of phase with rainfall. This is as a result of the fact that after rainfall the soil is able to retain most of the moisture and gradually transmit some to the vegetation as the season progresses. Thus, soil moisture is widely recognized as a vital element that connects precipitation and vegetation^52^. According to Nicholson *et al*.^26^, researches on NDVI in connection to soil moisture would transmit to a more detailed knowledge of the environmental limitations on vegetation growth changes. The set back to this is the non-availability of soil moisture data over the data sparse Africa countries.

### Coefficient of Variation (CV) in NDVI and Climate elements

**Figure 13:**
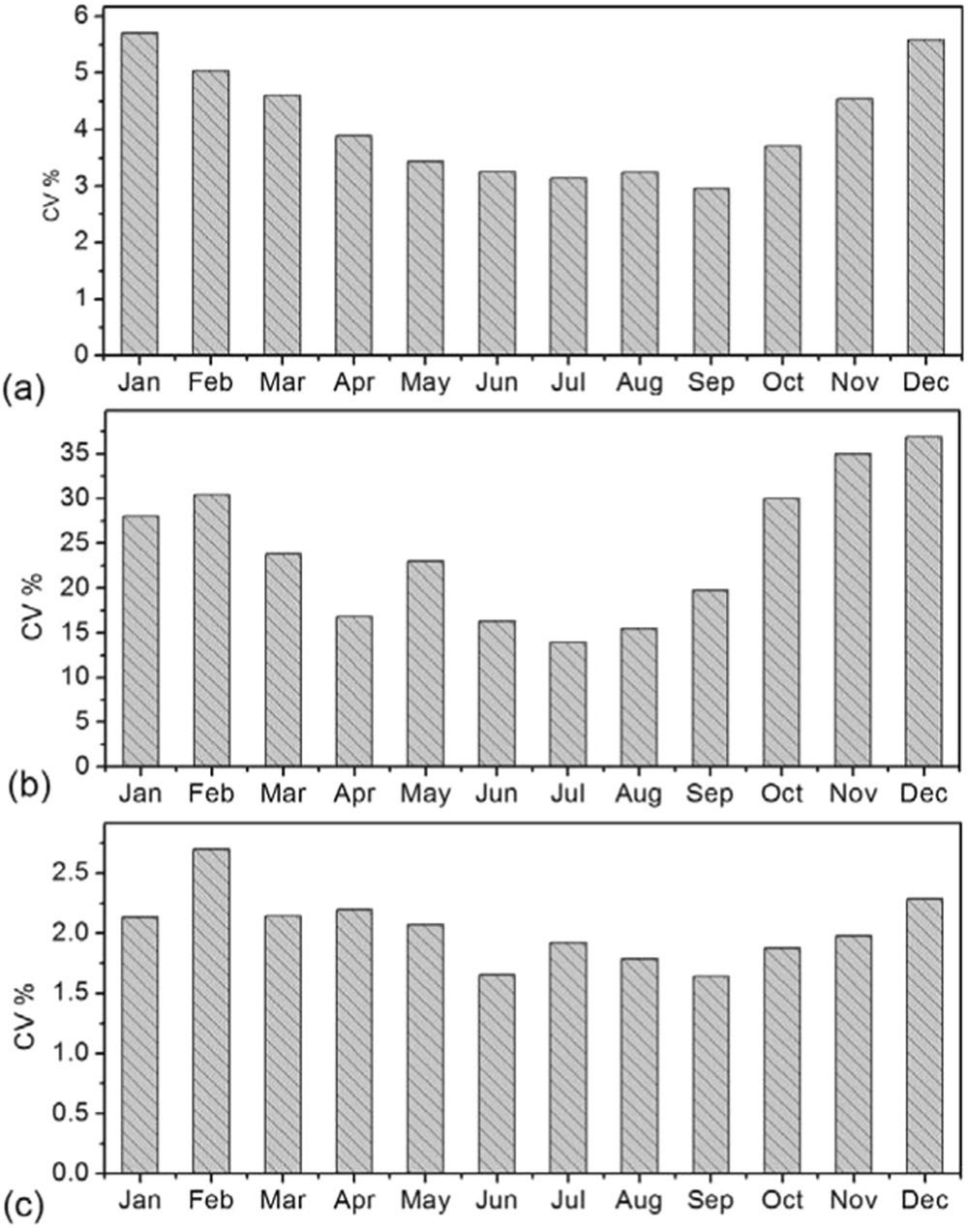
Coefficient of Variation for (**a**) NDVI, (**b**) Precipitation (mm/month) and (**c**) Temperature (o C) over East Africa from 1982 to 2015.

The concept of CV was adopted to assess NDVI variability analogous to the mean monthly values, while the standardized NDVI values also known as Z-scores were applied to expunge the NDVI seasonality and to measure the shift of NDVI with respect to the mean-monthly values^45^. The seasonal CV shown in Fig. 13 was analyzed separately to show significant patterns in vegetation variability across the seasons. In Fig. 13, NDVI, precipitation and temperature showed high CV of 5.7, 36.9 and 1.6% in January, December and September, respectively. The CVs of NDVI and precipitation are in the end of the short rainy months as expected except temperature. For low CV, NDVI, precipitation and temperature indicated 3.0, 13.94 and 2.7% in September, July and September, respectively. The low CVs for NDVI, precipitation and temperature fall in between the two seasons.

Also, from Fig. 13(a), the CV of NDVI during the DJF season was highest and lowest during the JJA season over-lapping into September, and steadily increasing up to the end of the SON season before decreasing at the end of DJF season. For precipitation and temperature, the DJF season shows a reduced CV in January compared to December and February. In Fig. 13(b), low CV in April is as a result of peak rainfalls over the region and high CV in March and May compared to April describes the variation in precipitation during end of long rainy season. Overall, precipitation shows highest CV followed by NDVI then temperature (Fig. 13). Both NDVI and climate elements displayed an increased CV from September to December, which suggests high vegetation and climate dynamics during the short rainy season. Spatially, NDVI variability increases as one move into the dry land areas with a CV of >50% having been observed in those areas during the long and short rainy season (Fig. 13). The spatial CV appears to show a meaningful idea about vegetation dynamics over the region even though the values are obtained for two different seasons over the last three decades.

**Figure 14:**
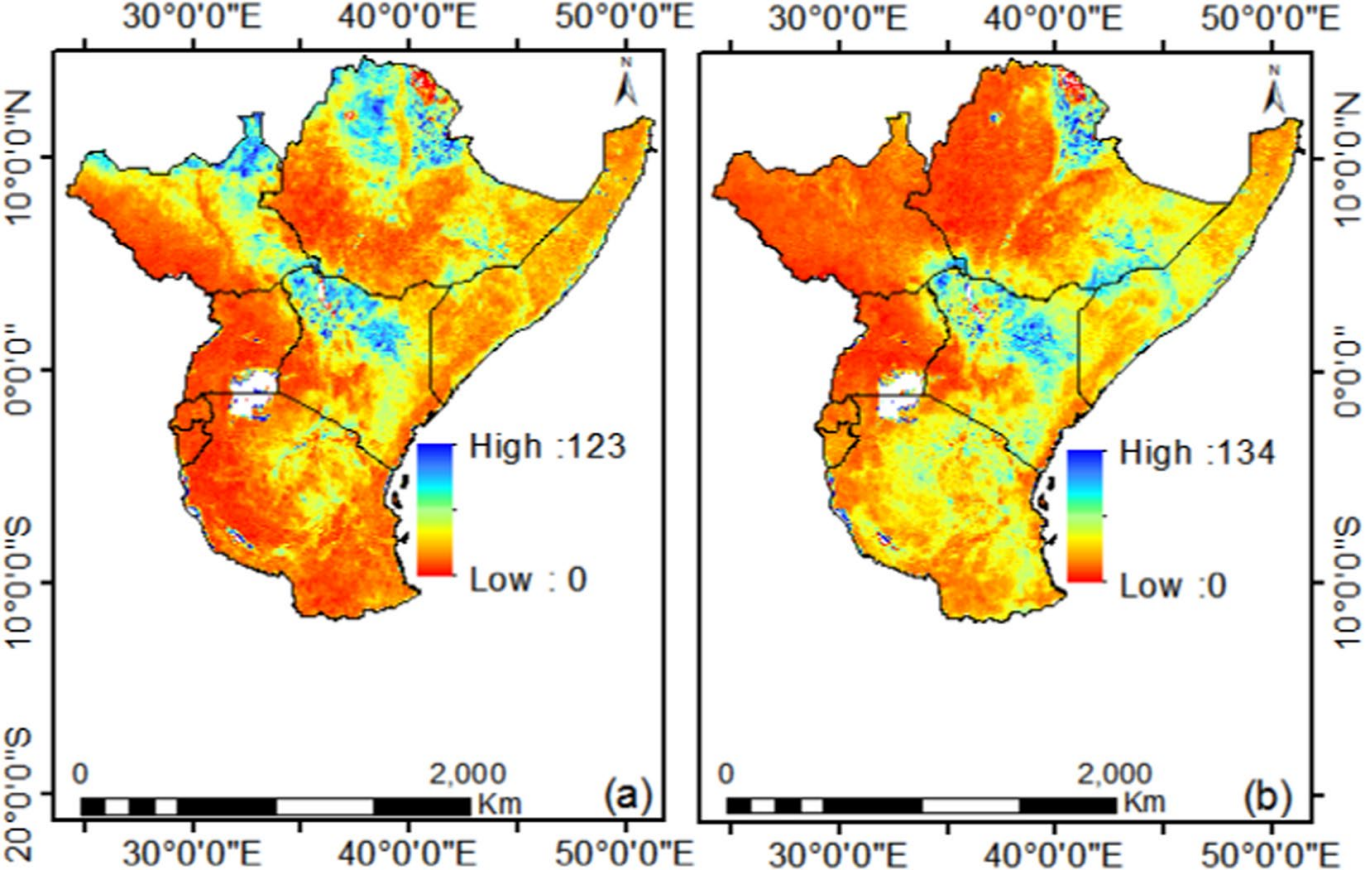
Spatial Coefficient of Variation in NDVI during the (**a**) Long and (**b**) Short rainy season from 1982 to 2015.

The spatial CV indicates that the mechanism of variation is different for the two seasons (Fig. 14). Agriculturist and Pastorist occupy areas that have very favorable climate conditions such as temperature and precipitation patterns which help them to develop. In Africa where Pastorist travel long distances around the grazing corridor, livestock production and maintenance is normally affected by climate variability. Both drought and flooded plains are not ideal for livestock rearing and any effect to vegetation caused by changes in climate affects both the farmer as well as animal. Therefore any change in the climate of such areas can have a lot of effects on the plant community including the animals existing there. They are most affected by the high variability as they rely more on rain fed agriculture and other natural resources affected by climate change over the region^7^.

## Conclusion

The 34-year remote sensing data of NDVI from the AVHRR sensors over the surface vegetation of East Africa was assessed in detail to study significant patterns in NDVI changes in response to climate change and variability. The results presented that the spatiotemporal vegetation productivity and variation over East Africa vary in different areas and time frames. The linear regression model A and piece wise regression model B are robust for detecting gradual and break points in mean NDVI and NDVI standard deviation, respectively. The negative trends observed in NDVI standard deviation after the BP is as a result of the improved vegetation greening from below normal conditions from 1980s and 1990s to above normal conditions in 2000s and 2010s. Results shows that before the BP, the mean annual NDVI (NDVI std. dev.) increased at 14.5 × 10^−4^/yr (3.96 × 10^−4^) and decreased significantly for NDVI (NDVI std. dev.) at −9.64 × 10^−4^/yr (−5.97 × 10^−4^) after the BP. Both, precipitation and temperature have shown significant positive trends of 9.7 × 10^−2^/yr and 2.7 × 10^−2^/yr respectively over the study region from 1982 to 2015. The occurrence of different spatial patterns for break and gradual changes with systematic variations in trends indicates the complexity in vegetation growth changes and a nonlinear relationship of vegetation to climatic changes and other non-climate drivers.

NDVI showed positive (negative) relationship with precipitation (temperature) both in the long and short rainy season. Also, all the two seasons, temperature is inversely related with precipitation. The distribution of NDVI pixels in long and short rainy seasons are unimodal and bimodal respectively. The same relationship between NDVI and climate parameters is observed using spatial data records. NDVI correlates differently with precipitation/temperature during the long and short rainy seasons. This suggests that, the moisture source in each of the season has an influence on the vegetation dynamics over East Africa. For NDVI values of the total vegetated area of East Africa, about 47.0 and 49.1% pixels are increasing and decreasing respectively while less significant amount remain unchanged from 1982 to 2015. The long-term decrease of vegetation as indicated by NDVI is a sign of degradation.

For the phenology metrics, results showed that the long growing season starts in March, peaked in May and in July while the short growing season starts in September, peaked in November and ends in December. In all, NDVI shows lags with precipitation in both the long and the short rainy and this explains the fact that while rainfall stops vegetation activity persist before returning to its dormant condition. Also, the study uncovered some links between NDVI and ENSO during most of the El-nino and La-nina years, specifically 1992/93 and 1997/98. The pattern of high (low) ENSO anomalies in 1998/97 (1992/93) corresponded with high (low) NDVI and rainfall values. The NDVI values during the 1997 (1998) are 1.3% (3.5%) above normal and 1.4% (0.4%) below normal (slightly above normal) in 1992 (1993). The above-normal NDVI was a result of excessive precipitation in 1997 which spilled up into 1998. Specifically, the damage caused by ENSO to vegetation is transferred via precipitation. The detected weak link between mean NDVI and ENSO suggests that there may be a weak teleconnection between NDVI and ENSO. The effect of ENSO on NDVI series is pronounced when vegetation is considered in seasons before actual months, suggesting a time lag between them.

## Supplementary information

Supplementary
