# Supplementary figures and images for: Assessment of climate impact on vegetation dynamics over East Africa from 1982 to 2015
Source: Sci Rep. 2019 Nov 14;9:16865. doi: 10.1038/s41598-019-53150-0 (PMC6856068; doi:10.1038/s41598-019-53150-0)

# Appendix I


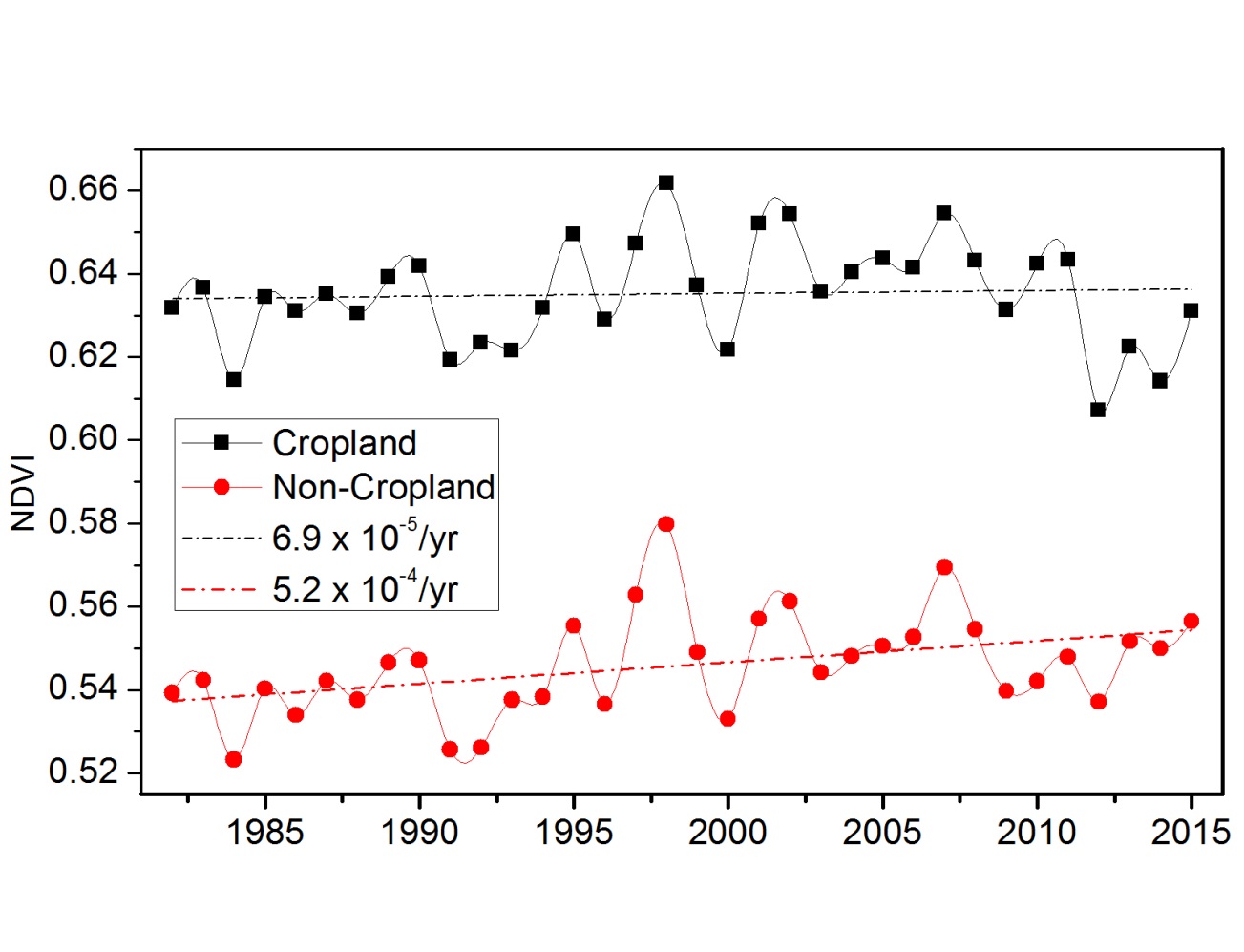

Supplement: Supplementary file 1 — Supplementary [file 41598_2019_53150_MOESM1_ESM.docx]
